# Patient, Caregiver, and Clinician Perspectives on Core Components of Therapeutic Alliance for Adolescents and Young Adults With Advanced Cancer: A Qualitative Study

**DOI:** 10.1001/jamanetworkopen.2023.28153

**Published:** 2023-08-09

**Authors:** Rosemarie Mastropolo, Andrea Altschuler, Katharine E. Brock, Mallory Casperson, Chun R. Chao, Lauren Fisher, Katie A. Greenzang, Lawrence H. Kushi, Joshua R. Lakin, Anna Lefebvre, Corey M. Schwartz, Dov M. Shalman, Catherine B. Wall, Lori Wiener, Jennifer W. Mack

**Affiliations:** 1 Department of Pediatric Oncology, Dana-Farber Cancer Institute, Boston, Massachusetts; 2 Division of Research, Kaiser Permanente Northern California, Oakland; 3 Department of Pediatric Oncology, Emory University and Children’s Healthcare of Atlanta, Atlanta, Georgia; 4 Divisions of Pediatric Oncology and Palliative Care, Cactus Cancer Society, Oakland, California; 5 Department of Research and Evaluation (C.R.C.), Kaiser Permanente Southern California, Pasadena; 6 Division of Population Sciences, Dana-Farber Cancer Institute, Boston, Massachusetts; 7 Department of Psychosocial Oncology and Palliative Care, Dana-Farber Cancer Institute, Boston, Massachusetts; 8 Division of Medical Oncology, Kaiser Permanente Northern California, Oakland; 9 Department of Palliative Care, Kaiser Permanente Southern California, Pasadena; 10 Pediatric Oncology Branch, National Cancer Institute, Bethesda, Maryland

## Abstract

**Importance:**

The patient-clinician therapeutic alliance is an important aspect of high-quality cancer care. However, components of the therapeutic alliance in adolescents and young adults (AYAs, aged 12-39 years) with cancer have not been defined.

**Objective:**

To identify components of and barriers to the therapeutic alliance between AYAs, caregivers, and clinicians from the perspective of all key stakeholders.

**Design, Setting, and Participants:**

In this qualitative study, semistructured in-depth interviews with patients, caregivers, and clinicians were conducted from 2018 to 2021 with no additional follow-up, with content analysis of resulting transcripts. Participants were recruited from Dana-Farber Cancer Institute, Kaiser Permanente Northern California, Kaiser Permanente Southern California, and an online cancer support community (Cactus Cancer Society). Eligible participants were English- or Spanish-speaking. Eligible patients were aged 12 to 39 years with stage IV or recurrent cancer. Eligible caregivers cared for an AYA living with advanced cancer or one who had died within 5 years. Eligible clinicians routinely provided care for AYAs with cancer.

**Main Outcomes and Measures:**

Perspectives on therapeutic alliance.

**Results:**

Interviews were conducted with 80 participants: 23 were patients (48% were female; 78% were White), 28 were caregivers (82% were female; 50% were White), and 29 were clinicians (69% were female; 45% were White). The mean (SD) age of patients was 29 (7.3) years. Interviews identified 6 components of therapeutic alliance: (1) compassion; (2) sense of connection; (3) clinician presence; (4) information sharing; (5) shared goals; and (6) individualization of care. While some domains were represented in prior models of therapeutic alliance, a unique domain was identified related to the need for individualization of the approach to care for AYA patients and their caregivers. Interviews also identified potential barriers to building the therapeutic alliance specific to the AYA population, including managing discordant needs between patients and caregivers and communication challenges at the end of life.

**Conclusions and Relevance:**

This study identified core components and barriers to building therapeutic alliance in the AYA advanced cancer population from the perspective of all the key stakeholders in the relationship. A novel component highlighting the need for individualization was identified. This model enables a deeper understanding of how to build therapeutic alliance in the AYA advanced cancer population, which may guide clinician training and facilitate improved care for this vulnerable population.

## Key Points

**Question:**

What do adolescents and young adults (AYA) with cancer, their families, and their clinicians identify as important aspects of the therapeutic alliance in end-of-life care?

**Findings:**

In this qualitative study including 80 participants, interviews with 23 AYAs, 28 caregivers, and 29 clinicians identified 6 components of therapeutic alliance. The components were compassion, sense of connection, clinician presence, information sharing, shared goals, and the importance of individualized care centered on the needs of the patient and their family.

**Meaning:**

Results of this study suggest that the core components to building therapeutic alliance may guide clinicians in their approach to partner with AYA advanced patients with cancer and their caregivers to improve end-of-life care in this vulnerable population.

## Teaser

This qualitative study assesses components of the ideal therapeutic alliance between adolescents .and young adults with advanced cancer, their caregivers, and their clinicians using in-depth interviews and identifies barriers to building therapeutic alliance in this population.

## Introduction

Nearly 90 000 adolescent and young adult (AYA) patients are diagnosed with cancer in the US each year, and cancer is the leading disease-related cause of death in AYAs.^1^ This population is at risk for inferior quality cancer care,^2,3,4,5,6,7,8,9,10,11,12,13,14,15,16^ in part due to inhabiting a vulnerable and transitional life phase with unique social, educational, employment, and family concerns.^3,4,16^ End-of-life (EOL) care can be particularly fraught for this population. A tenet of high-quality EOL care is a therapeutic physician-patient relationship,^17,18^ including in AYA patients with cancer.^19^

A strong therapeutic alliance, defined as the collaborative bond between clinicians and patients, has been shown to be an important independent factor in cancer patient care outcomes, quality of life, caregiver well-being, and caregiver bereavement outcomes.^20,21,22,23,24,25^ While evidence is largely cross-sectional without proven causation, among adult patients with cancer, a strong therapeutic alliance was associated with improved emotional acceptance of terminal illness, decreased existential distress, and decreased intensive interventions at EOL.^25^ Among AYAs, a stronger therapeutic alliance, measured with a tool designed for adults with cancer, was associated with improved patient and family psychosocial outcomes including quality of life, perceived social support, illness coping,^21^ finding meaning in illness, and peace of mind.^26^ A positive oncologist-patient relationship has also been found to be protective against suicidal ideation in AYA patients with cancer.^20^ Notably, the importance of therapeutic alliance extends beyond the patient-oncologist dyad to the caregivers of patients as well; the perception of a strong therapeutic alliance between adult patients with cancer and their oncologists prior to patient death was associated with enhanced social function and improved physical and psychological health in bereaved caregivers.^22^

Despite the importance of therapeutic alliance in patient and caregiver outcomes, we lack a cohesive definition of the components of the therapeutic relationship in the AYA population. A prior study of clinicians of AYAs proposed that therapeutic alliance encompassed human connection, empathy, presence, partnering, inclusivity, humor, and honesty.^27^ However, these data were compiled from the clinician perspective alone, without input from patients or caregivers as key stakeholders. We therefore sought to define components of the ideal therapeutic alliance between AYAs with advanced cancer, their caregivers, and their clinicians using in-depth interviews, and to identify barriers to building therapeutic alliance in this population.

## Methods

### Study Design and Participants

This study follows the Consolidated Criteria for Reporting Qualitative Research (COREQ) reporting guideline for qualitative research, detailed in eTable 1 in Supplement 1 with further details on the primary study methods.^28,29^ Participants were recruited between December 2018 and January 2021 for a qualitative interview study of AYAs with advanced cancer, caregivers, and clinicians of AYAs with cancer. The study was approved by the institutional review boards of participating sites: Dana-Farber Cancer Institute, Kaiser Permanente Northern California, and Kaiser Permanente Southern California. Signed informed consent, which included the use of quotations in publications, was obtained from all participants. The goal of the original study was to identify potential priority domains of high-quality EOL care from each stakeholders’ perspective.^28^ While not a focus of the interview questions, in the initial work, therapeutic alliance emerged as a strong priority for AYAs and caregivers. This secondary analysis was undertaken to define the components of the therapeutic alliance for AYAs and their caregivers.

Patients and caregivers were recruited from the 3 participating sites and an online support community, Cactus Cancer Society (formerly Lacuna Loft). Eligible patients included English-speaking or Spanish-speaking AYAs aged 12 to 39 years with advanced cancer (defined as stage IV or recurrent cancer), who were enrolled only if a readiness assessment indicated willingness to discuss EOL issues.^28,30^ The age range included patients defined as AYA by the World Health Organization (aged 12-24 years) and the National Cancer Institute (aged 15-39 years). Eligible caregivers included those of AYAs living with advanced cancer or who died within the past 5 years. While some patient-caregiver dyads participated, most patients and caregivers were enrolled independently. Eligible physicians, nurses, nurse practitioners, and psychosocial clinicians who care for AYAs with cancer were identified by each site’s investigators.

### Data Collection

Semistructured interviews were conducted in person (n = 25) or by telephone (n = 55) by trained interviewers using interview guides developed by the research team. The patient interview guide included open-ended questions exploring prognostic awareness, care experiences, care priorities, and differences across the AYA age spectrum (eTable 2 in Supplement 1). Race and ethnicity were self-reported and included as demographic characteristics. A multidisciplinary advisory group reviewed the interview guide to ensure developmental appropriateness and sensitivity. Caregivers were asked what they felt was most important in the care of their AYA family member. Clinicians were asked to reflect on priorities identified through general experiences treating AYAs.^28^

### Analysis

Directed content analysis^31^ was performed to evaluate transcribed interview data using NVivo software, version-1.4 (QSR International) with initial coding schema described in Table 1. Secondary analysis was performed using grounded theory approach and deductive coding of the previously coded data to specifically assess themes regarding clinician relationships and therapeutic alliance until thematic saturation was reached within each participant group. To ensure interpretive consistency, 2 investigators (R.M., J.W.M.) collaboratively reviewed the coded content, identified themes, and synthesized the data across themes. Included quotations underwent light editing for clarity.

**Table 1.** Participant Characteristics

Characteristic	No. (%)
Participating patients (n = 23)	
Sex	
Male	12 (52)
Female	11 (48)
Age group at participation	
12-24 y	5 (22)
25-39 y	18 (78)
Self-reported race and ethnicity^a^	
Asian	0
Black	0
Hispanic	4 (17)
White	18 (78)
Other or unknown^b^	1 (4)
Participating caregivers (n = 28)	
Sex	
Male	5 (18)
Female	23 (82)
Self-reported race and ethnicity	
Asian	0
Black	4 (14)
Hispanic	2 (7)
White	14 (50)
Other/unknown	8 (29)
Patient vital status at time of interview	
Living	5 (18)
Deceased	23 (82)
Patient age group at participation	
12-24 y	14 (50)
25-39 y	14 (50)
Relationship to patient	
Parent	22 (79)
Spouse/partner	5 (18)
Sibling	1 (3)
Participating clinicians (n = 29)	
Sex	
Male	9 (31)
Female	20 (69)
Self-reported race and ethnicity^a^	
Asian	6 (21)
Black	3 (10)
Hispanic	5 (17)
White	13 (45)
Other or unknown^b^	2 (7)
Discipline	
Physician	15 (52)
Nurse or nurse practitioner	6 (21)
Social worker or psychologist	8 (27)
Specialty	
Pediatric oncology	5 (17)
Medical oncology	8 (28)
Pediatric hospice or palliative medicine	2 (7)
Medical hospice or palliative medicine	6 (21)
Pediatric psychosocial care	3 (10)
Medical psychosocial care	5 (17)

^a^ Race and ethnicity were reported from participant response to how they identified.

^b^ Patients reported race and ethnicity verbally during the interview with the following questions rather than by survey: “Do you consider yourself Hispanic/Latino?” “How would you describe your racial background”? [may suggest Asian or Pacific Islander, Black, Native American, White, or other].

## Results

Of 80 participants: 23 were patients (48% were female; 78% were White), 28 were caregivers (82% were female; 50% were White), and 29 were clinicians (69% were female; 45% were White) (Table 1). The mean (SD) age of patients was 29 (7.3) years. Caregivers included 22 parents, 5 spouses/partners, and 1 sibling; most were bereaved (82%). Participating clinicians included 15 physicians, 6 nurses/nurse practitioners, and 8 social workers/psychologists.

We identified 6 domains that contributed to therapeutic alliance for the AYA population based on the patient and family perspective, which were corroborated by the clinician perspective: (1) compassion; (2) sense of connection; (3) clinician presence; (4) information sharing; (5) shared goals; and (6) individualization of care. The Figure presents the conceptual model, and Table 2 expands the model with specific components.

**Figure.**
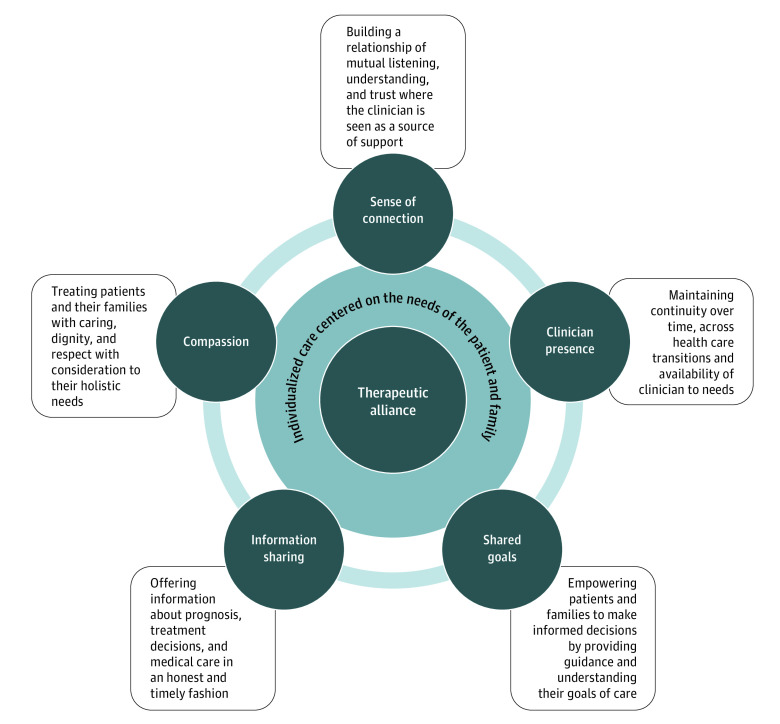
Conceptual Model of Components of Therapeutic Alliance in Adolescents and Young Adults With Advanced Cancer

**Table 2.** Components of Therapeutic Alliance With Relevant Interview Excerpts

Domain and relevant subdomain	Interview excerpt^a^
Compassionate care	
Dignity, respect and caring	BCG 29: They treated him like he was a friend. He wasn’t pitied […] they just treated him special. They had a really unique way of treating him like an adult […] Just every single person I came in contact with was so compassionate and just what we needed at that time, and it was just a good feeling.
Recognition as a whole person	Patient 136: I think it that it comes back to that sensitivity in that oncologists and the care team as a whole needs to talk to the patient and be like, “ok, what are your plans and how can we work around that?” […] because people have lives and they don’t necessarily stop just because you have cancer.
Sense of connection with clinicians	
Human bond and personal connection	BCG 50: I feel like the more people know about people’s personalities, that I feel like they’re going to better relate to them and care for them.
Listening and understanding	Patient 83: I think like for me it’s really important that as a patient I’m heard and involved, and […] respected.
Trust	Patient 138: I rely on my doctors and I trust their opinions.
Clinician presence	
Continuity	BCG 6: I know that having the same group of people caring for him – I think that helped him, because he […] developed that little bit of connection with the people.
Presence across transitions	BCG 29: I mean, [oncologist] treated [patient] like he’d known him forever. And he was by his side during times when […] it wasn’t even required that he be there. And [patient] was so grateful, and the way he was treated […] You know, I think of these things every day and I’m so grateful.
Availability	Caregiver 40: So the access is extremely important […] He feels like he can get to them should anything occur and I think that makes him feel a great deal of comfort.
Information sharing	
Honesty	Caregiver 25: I think always talking with the truth […], being direct and straight forward saying what she has and what’s really going to happen if we don’t do this and that. That helps us to give her the better care.
Willingness to address difficult topics	BCG 153: Dr [oncologist] came in and said one day, I think we need to have a discussion about plan B. And she took [patient name] through how she […] would want to die if she didn’t win […] which is something that I was doing the opposite of and it was causing huge concern for [patient] […] Dr [oncologist] put that to rest. Just with that, she just knew how to lead into that; Caregiver 25: Sometimes they try not to hurt the family and don’t say things that are gonna happen, but it’s preferred to tell the family so we can be alert and we can give her more care for her to be more comfortable.
Involvement in conversations tailored to patient’s and caregiver’s needs	BCG 150: For the team it was kind of difficult because they want to be forthcoming, but, sometimes being forthcoming wasn’t good for her and would set her back emotionally, mentally. So we kind of worked out a way between the doctors and myself on what to tell her and what not to tell her, so that she could continue to try to remain positive […] I just probably would suggest that they kind of coordinate with the parent how best to tell certain things to the child; Caregiver 27: Just keeping him informed. He needs to know. Good. Bad. Whatever. Like he needs to know everything. That makes him more comfortable. And I think it’d be really important for them to realize that they just need to tell him how it is. All the time; Clinician 69: I mean some patients really prioritize not talking about any of this, that they really just want their parents to call the shots and they just prioritize kind of not knowing, or not being involved, or having their parents communicate to them, not having to have direct conversations with providers.
Shared goals between the patient, caregiver and clinician	
Clarity about choices and expected outcomes	Patient 83: I definitely want to […] know all of the options available, including like new options. […] I want to be able to have open dialogue with my providers where they’ll be honest about the options on the table and able to really talk those through with me.
Focus on the patient’s and caregiver’s values and goals	BCG 54: Dr [oncologist] knew from the very beginning what was important to us. And she so graciously always said, quality of life is just going to support where we’re at. She always knew if I went off the track and started thinking about things, she would bring me back […] it was always our decision. We decided we were in the driver’s seat. She would give us the facts […] and we made the choices. […] I can’t speak volumes of the open communication and identifying what is important to the child and the family, and then trying to meet their needs.
Respect for the decision-making capacity and autonomy of patients and caregivers	Clinician 26: We never tell someone what they should or shouldn’t do, but just help them think through the pros, the cons, the risks, the benefits, and what that might mean based on what they’ve articulated their goals are.
Individualization of care	Caregiver 27: I feel like that’s super personal. At least when I talk to other patients […] some patients’ families want to know everything, want to talk about it. Others really just don’t – like just tell us what you’re doing and do it […] if there was some training on like how we could read people, that would be awesome; Clinician 67: I think there’s a lot of advanced care planning that can be challenged by having to figure out how the system looks like for each individual family – how they communicate, how they talk about illness, how they talk about what to expect. Whether or not they’re a family who can tolerate kind of talking about it far in advance, or sometimes not so far in advance. And what that looks like and what that means is often the biggest challenge.

Abbreviation: BCG, bereaved caregiver.

^a^ When needed to describe a range of perspectives, more than 1 quotation may be included. Each domain did not necessarily have subdomains.

### Compassion

Compassion included 2 relevant subdomains: being treated with dignity, respect, and caring and being valued as a whole person, separate from their disease. Patients and caregivers emphasized the need for clinicians to respect them and their experience. This was achieved when clinicians prioritized patients’ perspectives and offered choices that support their dignity. Participants also wanted their lives and goals outside of their medical care to be acknowledged and accommodated when possible: “I think they should understand that I have all of these plans lined up and all of these goals” (patient 84).

### Sense of Connection

Within the domain of connection, relevant subdomains included experiencing a sense of personal connection with their clinicians, feeling heard and understood, and trusting their clinician. Participants identified the importance of seeing their clinician as a trusted source of support, noting this trust engendered a sense of security and helped reduce anxiety: “It’s really important that there’s somebody who you trust a lot. I trust her judgment calls so much. That’s a huge comfort to me” (patient 83).

### Clinician Presence

Patients and caregivers of AYAs valued maintaining continuity with clinicians over time and over health care transitions such as admissions, enrollment to hospice, and after a patient’s death. Participants identified that a lack of clinician support during transitions could lead to feelings of abandonment for patients and families. Clinician presence also encompassed being available and accessible to patients and their families for concerns that may arise outside of visits: “That’s really important to me that I have a good relationship with my doctor team. I feel that safety net of knowing that anytime I had a new symptom, I could go in to get it checked out” (patient 138).

### Information Sharing

Within the domain of information sharing, participants emphasized the importance of clinicians offering information about prognosis, treatment decisions, and medical care in an honest and timely fashion, and in a way that was appropriate for the patient and the family. While many participants valued clinician honesty to allow for informed decision-making, the ideal timing and level of involvement of patients and their families in these conversations varied between participants. While some clinicians noted that discussing difficult information related to EOL with patients and caregivers might challenge the therapeutic alliance, many patient and family participants wished these conversations occurred earlier to better inform care decisions: “The only thing I wish could have done different is discussed end-of-life procedures at the very beginning. It would have made the end easier because we all would have been clear on what she wanted” (bereaved caregiver 151).

### Shared Goals

Participants highlighted shared goals between the patient, family, and clinician as an important component of therapeutic alliance. To enable shared goals, patients and caregivers recommended that clinicians explain potential options, set expectations about possible outcomes, and provide recommendations when appropriate to empower patients and families to make informed decisions aligned with their values. Clinicians also described a need to understand the patient’s values and respect their autonomy, even when their decisions conflicted with the clinician’s perspectives: “Listen more to what they want. Ask more questions because sometimes they won’t tell you, but if you ask, they will answer those questions. We need to take their opinions more seriously and honor those opinions more. My idea of what’s right for them may not be their own idea of what’s right” (clinician 108).

### Individualization of Care

In each of these domains, participants emphasized the need for individualization of care. This was especially salient for the domains of information sharing and shared goals. To build a strong therapeutic alliance, patients and family members recommended that clinicians recognize that each patient and family may have different values regarding the degree of information sharing, involvement in treatment decisions, and ultimate goals of care. Participants emphasized the importance of the clinical team identifying and adapting to their personal needs.

Within each domain, we identified no differences across participant type and patient age. The ideal approach to building relationships was not determined by age but rather needed to be individualized for each patient and caregiver: “There’s a lot of advance care planning that can be challenged by having to figure out how the system looks for each individual family–how they communicate, how they talk about illness, how they talk about what to expect. Whether they’re a family who can tolerate talking about it far in advance, or not so far in advance. What that looks like is often the biggest challenge” (clinician 67).

### Barriers to Building Therapeutic Alliance

In addition to these components of therapeutic alliance, participants identified important barriers to strong relationships between AYAs, caregivers, and clinicians (Table 3). One noted challenge involved managing discordant needs between patients and caregivers. Patient and caregivers expressed frustration with navigating care across complex health care systems and the resulting communication breakdowns, which could also undermine the clinician-patient-caregiver therapeutic alliance. Finally, clinician reluctance to initiate EOL conversations creates barriers to the therapeutic alliance. For example, clinicians expressed discomfort discussing EOL care with AYA patients given limited exposure to the AYA population and a lack of training on leading these conversations with AYAs. Clinicians also worried about the optimal timing for EOL conversations with concern for loss of hope among patients and their families, and they feared that difficult news could erode trust in the clinician or harm their relationship with the patients. Fear of the emotional burden of these conversations on both patient-caregiver dyads and the clinicians themselves was commonly identified as a barrier to initiating EOL conversations. However, avoidance of these challenging conversations had potential to impair the therapeutic alliance rather than preserve it.

**Table 3.** Barriers to Therapeutic Alliance With Relevant Interview Excerpts

Domain and relevant subdomain	Interview excerpt^a^
Conflict between managing patient’s needs and caregiver’s needs	Clinician 66: I guess I see one challenge is who is making the primary decisions, whether it’s the patient, him or herself or the parents. BCG 28: There’s not a lot of elasticity around the way, if we’re talking about young adults and the parents are there in the room, there’s not a lot of elasticity around the parents’ reaction because the medical team is focused on the patient which is as it should be. But for me at times the honesty was difficult, but it was vital to [patient’s name].
Fragmentation of care	Patient 83: A thing that I just continually find frustrating about treatment is when it seems like there’s sometimes breakdowns in communications between the different departments or sometimes between like the member services and the medical offices, and the patients themselves are often put in the place, put in the position of having to relay information back and forth, which we’re not very good at.
Clinician avoidance of end-of-life communication	
Discomfort communicating with young patients	Clinician 102: I think another barrier toward care is there’s a little bit of discomfort among treating younger patients for many doctors, I think that sometimes can be a barrier toward addressing some of the issues that we can, we’re comfortable with talking about with adults. So, things like end of end-of-life planning, discussing goals of care, looking into noncurative situations. I think unfortunately, those are probably delved into less than we should with the younger population.
Lack of training	Clinician 64: I think supporting providers in developing language to bring it up sooner in a way that doesn’t feel like we’re giving up on you— again, kind of respecting that population tends to be 100 percent committed to aggressive therapy until they’re not.
Concern for loss of hope	Clinician 67: I think that there is an overwhelming sense [that…] talking about these things upfront, during treatment strategies, or when we’re still exploring curative intent options – can feel like we’re stripping people of their hope, or we’re going to confound the situation with uncertainty. Or that people won’t be able to kind of tolerate those conversations, and we’re going to ruin our relationships with them.
Fear of ruining therapeutic bond with patient	Clinician 103: And then just trying to prepare them for the end and trying to get them to think about it and what they would want at the end, because not a lot of people under the age of 40 even want to spend any time thinking about it….So we are trying to be the person who gets them to focus on it while still having them not hate you for it. That’s pretty difficult.
Emotional toll on patients, caregivers and clinicians	Clinician 105: I think those conversations are very difficult, and so, I think whether it’s providers not making space for those conversations or patients not being emotionally able to have those conversations or family members impeding those conversations, I think that all those are possible reasons why we don’t- why we might not be doing a great job having those conversations.

Abbreviation: BCG, bereaved caregiver.

^a^ When needed to describe a range of perspectives, more than 1 quote may be included. Each domain did not necessarily have subdomains.

## Discussion

Through content analysis of qualitative interviews, we identified 6 important components that contribute to therapeutic alliance for AYAs with cancer, their caregivers, and their clinicians: compassion, sense of connection, clinician presence, information sharing, shared goals, and individualization of care. Prior conceptual frameworks for therapeutic alliance in pediatric and adult cancer care are few and do not include patient or caregiver input.^27,32,33^ This study included the perspectives of patients and caregivers as key stakeholders in defining the components of the therapeutic alliance in the AYA population. Our findings highlighted aspects of personal connection and communication previously identified as integral components of high-quality EOL care^17,18,19^ and the physician-patient relationship,^25,27,34,35,36,37^ along with aspects not previously reported such as the need for individualization of care. This novel conceptual model may serve as a framework for future research and intervention development to guide clinicians in their efforts to partner with AYA patients and their caregivers to build therapeutic relationships, especially during difficult times such as EOL care, in a way that meets patient and family needs.

A unique aspect of therapeutic alliance identified in this study was the need for individualized, patient-centered care. While this area has not previously been included in models of therapeutic alliance,^25,27,34^ prior work regarding AYA’s preferences for treatment decisional involvement in cancer care corroborates the need for individualization for each patient-caregiver dyad and for each unique decision, given that preferences for involvement can change with time and circumstance.^38^ This need for a flexible approach may reflect both the transitional nature of this life stage for AYAs, characterized by a spectrum of needs that depend on each individual’s developmental stage and maturity, and the triadic nature of the patient-caregiver-clinician relationship, which necessitates assessing and addressing the needs of both the patient and caregivers with a personalized approach. However, as themes were consistent across participant type and patient age, it suggests the ideal approach to building relationships, communication, decision-making, and EOL care for the AYA population is not determined by patients age, but rather needs to be individualized for each patient and caregiver to best meet their needs and build a strong therapeutic alliance. By understanding this need, we hope to build on existing literature to develop an approach to help clinicians engage AYAs and their caregivers.^39^

We also identified potential barriers to developing a strong therapeutic alliance. The triadic nature of the relationship adds complexity in building rapport with patients and their caregivers, given the potential for conflict or disparate needs between patients and caregivers, confusion over the optimal role for caregivers, and concerns about tailoring communication for each participant.^40,41,42,43,44^ This becomes especially challenging when considering the medicolegal concerns regarding who is the primary decision-maker for younger AYAs as well as needs among AYA patients to navigate growing independence.^44,45^ Clinician training on how to approach a patient-caregiver dyad to address their individual concerns may alleviate some of the conflict and support unique therapeutic relationships between each member of the triad.

Clinicians cited challenges communicating about issues like prognosis and EOL priorities with AYAs, in part because of fear of harming the relationship. Clinicians’ worries about loss of hope and the negative impact on the therapeutic bond have previously been noted in literature as barriers to communication, particularly if a difficult conversation occurs early in the treatment course before a patient-clinician relationship is established.^46,47,48,49,50^ Despite clinicians’ concerns regarding causing emotional distress and diminishing hope, there is evidence that patients and their families often desire clear and honest prognostic information even when the prognosis is poor,^25,46,51,52,53^ and that detailed prognostic information is associated with higher communication-related hope, peace of mind, trust in the oncologist, and decreased emotional distress and caregiver decisional regret.^25,46,53,54,55,56,57^ AYA patients value timely discussions regarding EOL care, with most preferring earlier disclosure of prognosis to better inform their decisions.^51,52,58^ Clinician discomfort communicating with AYAs specifically may be related to limited exposure within their practice; medical practitioners likely have greater experience and training on holding EOL conversations with older adults and pediatric clinicians with adult parents of their patients, leaving clinicians feeling underprepared in addressing the unique AYA population. Advance care planning tools that facilitate such discussions alleviate patient anxiety, improve congruence in understanding of patients’ preferences, decrease caregiver decisional regret and could relieve clinician discomfort and concerns of lack of training on broaching EOL conversations with AYAs.^30,38,51,59,60,61,62^ In our study, patients and caregivers echoed this value for information sharing with emphasis on clinician honesty and willingness to address difficult topics. Our data suggest this approach must be individualized based on patient and caregiver preference to allow for tailored involvement of the AYA, but even so, it is important to recognize that many of the clinician-identified worries regarding communication barriers are not supported by patient- and caregiver-reported data. Evidence supports timely, detailed, and honest communication to AYA patients and their families, which may help to address the uncertainty inherent in cancer care and empower patients and their families to engage in decision-making and care with better understanding.^47^ Ultimately, patients and caregivers considered this kind of shared work to be a core goal of a successful therapeutic relationship, not an impediment to it.

### Limitations

Study limitations include limited racial and ethnic diversity among participants, for which the offering interviews in English or Spanish was a potential factor. Values for the therapeutic relationship may be culturally determined; future work in a more racially, ethnically, and culturally diverse sample will be important. Fewer patients in the youngest age range were included, due to difficulties surrounding in-person interviews during the early phase of the COVID-19 pandemic. To ensure the experience of the younger population was captured, we included more caregivers of adolescents and pediatric clinicians. Notably, the content from interviews across younger and older patients was consistent regardless of age. Finally, patients and caregivers who elected to participate in the study may have different perspectives than those who declined to participate. Most caregivers were female and bereaved, and their experience may not be representative of male or nonbereaved caregivers. We included caregivers of patients who declined and clinicians to help represent a broader range of experiences.

## Conclusion

In our initial work to identify aspects of EOL care quality among AYAs, the patient-caregiver-clinician therapeutic alliance emerged as a critically important aspect of care among AYAs with advanced cancer. We therefore sought to understand in depth the components most valued by patients and caregivers. This quality improvement study identified core components and barriers to building therapeutic alliance in the AYA advanced cancer population from the perspective of all key stakeholders in the relationship, including a novel component highlighting the need for individualization that adds to the current literature. These findings offer a framework for clinician education and future research on interventions to build, repair, and address barriers to therapeutic alliance in the AYA population to provide high-quality care for AYAs.

## Notes

Supplement 1. **eTable 1.** Role Descriptions in Use of Consolidated Criteria for Reporting Qualitative Studies (COREQ) Checklist **eTable 2.** Sample Patient Interview Questions

Supplement 2.
Data Sharing Statement
