# Supplementary material for: Patient, Caregiver, and Clinician Perspectives on Core Components of Therapeutic Alliance for Adolescents and Young Adults With Advanced Cancer: A Qualitative Study
Source: JAMA Netw Open. 2023 Aug 9;6(8):e2328153. doi: 10.1001/jamanetworkopen.2023.28153 (PMC10413170; doi:10.1001/jamanetworkopen.2023.28153)
Supplement: Supplement 1. — eTable 1. Role Descriptions in Use of Consolidated Criteria for Reporting Qualitative Studies (COREQ) Checklist eTable 2. Sample Patient Interview Questions [file jamanetwopen-e2328153-s001.pdf]

Supplemental Online Content

Mastropolo R, Altschuler A, Brock KE, et al. Patient, caregiver, and clinician perspectives on core components of therapeutic alliance for adolescents and young adults with advanced cancer. *JAMA Netw Open.* 2023;6(8):e2328153. doi:10.1001/jamanetworkopen.2023.28153

eTable 1. Role Descriptions in Use of Consolidated Criteria for Reporting Qualitative Studies (COREQ) Checklist

eTable 2. Sample Patient Interview Questions

This supplemental material has been provided by the authors to give readers additional information about their work.

© 2023 Mastropolo R et al. *JAMA Network Open*.

eTable 1. Consolidated criteria for reporting qualitative studies (COREQ) checklist

Domain	Item	Description
**Personal Characteristics**	Interviewer/facilitator	Interviews were conducted by 4 trained interviewers (J.W.M., B.V., G.R., I.J.)
	Credentials and occupation	One interviewer was a pediatric oncologist with medical doctorate and masters in public health degrees (J.W.M). The other interviewers were research assistants with baccalaureate degrees (B.V., G.R., I.J.)
	Gender	Interviewers were all female.
	Experience and training	J.W.M. is an experienced qualitative interviewer. Research assistants were trained on administration of readiness assessment and completed observations of interviews in addition to further training in conducting qualitative semi-structured interviews by J.W.M and L.W.
**Relationship with participants**	Relationship established	No interviewers had pre-existing relationships with participants. One interviewer (J.W.M.) is a pediatric oncologist and conducted limited interviews with medical oncology patients during interviewer training but was not involved in their care.
	Participant knowledge of the interviewer	Participants had no prior knowledge regarding the interviewer.
	Interviewer characteristics	One interviewer (J.W.M.) is a pediatric oncologist with research interest in therapeutic alliance and palliative care topics. The remaining interviewers were research assistants who had completed undergraduate degrees and were involved in a variety of clinical patient-oriented research studies.
**Theoretical framework**	Methodological orientation and theory	Directed content analysis using grounded theory approach and deductive coding was used to analyze the transcribed interview data.
**Participant selection**	Sampling	Purposive sampling was employed to ensure representation across the AYA age range, race/ethnicity, caregiver relationship and clinician type given the potential impact of these participant characteristics on their values and opinions.
	Method of approach	Eligible patients and caregivers were identified via clinic lists, administrative databases and/or referral from clinicians. An online advertisement on Cactus Cancer Society website allowed for self-referral of patient and caregivers. Eligible clinicians were

© 2023 Mastropolo R et al. *JAMA Network Open*.

		identified by investigators at each site. Eligible patients and caregivers were approached in-person or by mail with a letter describing the study and including an informed consent document. Signed informed consent was obtained from all participants.
	Sample size	We sought to recruit 20-30 of each participant type (patients, caregivers, and clinicians) with recruitment through thematic saturation with respect to priorities for end-of-life care in each group and with representation across the AYA age spectrum.
	Non-participation	Sixty individuals declined participation, including 32 patients (or caregivers of adolescent patients who declined on their behalf), 8 caregivers, and 20 clinicians. No eligible patients who were interested were excluded after completing the readiness assessment. No enrolled participants ended their interview early or dropped out of the study.
**Setting**	Setting of data	Interviews were conducted in-person for adolescent patients (aged 12-24 years old) at Dana Farber Cancer Institute and by phone for all other participants.
	Presence of non-participants	Non-participants were sometimes present for the interview at the discretion of the patient participant or for patients <18 years old, at the discretion of their caregiver. Patients and caregivers from one family had the opportunity to participate separately or together in the interview based on preference.
	Description of sample	Refer to Table 2 for participant demographics and to the Results section for important data which emerged.
**Data collection**	Interview guide	An interview guide was developed by the authors of the primary study and reviewed by the advisory group, which included a patient advocate, to ensure developmental appropriateness and sensitivity for all participants (eTable 1). This was not pilot tested prior to implementation.
	Repeat interviews	Participation ended once the initial interview was complete with no additional follow-up.
	Audio/visual recording	Interviews were audio-recorded, transcribed and reviewed for interview quality.

© 2023 Mastropolo R et al. *JAMA Network Open*.

	Field notes	Limited field notes were obtained during the interviews with focus on monitoring for signs of emotional distress.
	Duration	Interviews ranged from 14-79 minutes (median time 31 minutes).
	Data saturation	Thematic saturation with respect to priorities for end-of-life care in each group was achieved in the primary study. From this, relationships with clinicians emerged as a prominent theme within the parent study as core end-of-life priority domain. A secondary analysis of the data was performed to assess sub-themes related to clinician relationship to define the components of therapeutic alliance until thematic saturation was achieved. These specific sub-themes were commented on by 21/23 patients, 27/28 caregivers and 27/29 clinicians.
	Transcripts returned	Participation ended once the interview was complete with no transcript return.
**Analysis and Findings**	Description of the coding tree	For primary analysis, an initial coding schema was developed based on domains of high quality end-of-life care from the literature and a priori conceptual categories and applied by two investigators (J.W.M. and L.F.). Secondary analysis was performed using grounded theory approach and deductive coding to specifically assess themes related to clinician relationships and therapeutic alliance by two investigators (J.W.M. and R.M.).
	Derivation of themes	Themes were derived from the data.
	Software	NVivo software, version 1.4 was used for directed content analysis.
	Participant checking	Participant checking was not performed.
**Reporting**	Quotations presented	Illustrative quotations were presented in the Results and in Table 3 and 4 with appropriate participant identification.
	Data and findings consistent	There was consistency between the interview data and the findings presented.
	Clarity of major themes	Major themes from our analysis are reported in the Results section.
	Clarity of minor themes	Minor themes are unable to be fully assessed given the sample size of the study, especially considering the sub-groups within participant groups, and given this is a secondary analysis with the primary study not designed to elicit nuanced data regarding therapeutic alliance.

© 2023 Mastropolo R et al. *JAMA Network Open*.

© 2023 Mastropolo R et al. *JAMA Network Open*.

eTable 2: Sample Patient Interview Questions

**Introductory questions for all patients**	I would like to ask you some questions about what you think is most important to people your age with cancer if treatment is no longer working (and cure is not possible). That may not be the situation you are in, but we think as a young person with cancer, you can help us understand your perspective and what matters to young people in that situation.<br><br>I'd like to learn a little bit about your illness. Can you tell me what you believe is ahead for you in your illness? [*Probing questions if needed:* what do you think might happen to you because of your illness? How do you think your illness can affect you in your future? In what ways might your illness affect your future plans or goals?]
**Follow-up questions for patients who acknowledge a poor prognosis**	You just told me that you think about *[use patient's words.]* Some patients start to make plans for the care they would or would not like to receive if treatment doesn't work well. Is this something you have thought about?<br><br>*If **YES**, patient **HAS** thought about care plans:* What kinds of things have you thought about? Have you discussed your thoughts with anyone else? If so, who?<br><br>*If **NO**, patient **HAS NOT** thought about care plans:* As you think about your life right now, what things are most important to you? Are there things that are especially important to achieve or accomplish? Are there things that you especially value having in your life?<br><br>What about when you think about your medical care? What is most important to you in your care right now?<br><br>What about things that you would like to be sure do not happen to you, either in your life or in your medical care?<br><br>Now I am going to ask you an especially difficult question. You can tell me you want to move on and not answer it if you want. Okay? Most of us have "what if" conversations that come to our mind. We tend to have them when we are alone, or trying to sleep. If you were faced with a time when it seemed that you could die of your illness, can you imagine what might be most important to you at that time? Tell me about that. Can you imagine anything you might not want to happen to you? What else has gone through your mind?<br><br>These are hard things to talk about, but I'm grateful that you are sharing your thoughts. Are you okay for a few more questions?<br><br>Okay. Now I would like to talk about what we know about the experiences of young people with cancer who can't be cured, and who eventually die of their cancer. We know that, in their last few weeks of life, most of them experience a lot of medical care, like chemotherapy, time in the hospital or emergency room, and time in the intensive care unit. What do you think about that? Does that sound like the kind of care you would want, the kind of care you wouldn't want, or would it depend? Why?<br><br>We also know that many young people die in the hospital, although some die at home. Can you imagine what you would want? Why?<br><br>Are there any ways you would want to be sure that your life has had meaning?

© 2023 Mastropolo R et al. *JAMA Network Open*.

	We have talked about different priorities that you have, and also different priorities that other young people have. As you think about the many different things that are important to you, what do you think is most important for doctors and nurses to understand about you? If you could tell your doctor or nurse anything about what matters to you most, what would you tell him/her?<br><br>Is there anything else you would like for me to know about the things we have talked about? Are there other things that you would like to express about what might be important to people your age if they get to a point when treatment is no longer working?<br><br>Thank you for talking with me about these things. I know these are hard topics but I have learned so much from your perspective.
**Follow-up questions for patients who do not acknowledge a poor prognosis**	As you think about your life in general, what parts are most important to you?<br><br>What is most important to you in your care right now?<br><br>What about things that you would like to be sure do not happen to you, either in your life or in your medical care?<br><br>Now I would like for you to imagine something difficult- I would like for you to imagine that treatment was no longer working for you. I am glad that you aren't in this situation, but I am hoping you can help me understand what might be important to you if you were.<br><br>What parts of your life do you imagine might be most important to you if treatment were no longer working?<br><br>Some patients start to make plans for the care they would like to receive if treatment doesn't work well. Can you imagine what kinds of things might be important to you about your medical care if you were ever in that situation?<br><br>Now I am going to ask you an especially difficult question. You can tell me you want to move on and not answer it if you want. Okay? Most of us have "what if" conversations that come to our mind. We tend to have them when we are alone, or trying to sleep. If you were faced with a time when it seemed that you could die of your illness, can you imagine for me what might be most important to you at that time? Tell me about that. Can you imagine anything you might not want to happen to you? What else has gone through your mind?<br><br>These are hard things to talk about, but I'm grateful that you are sharing your thoughts. Are you okay for a few more questions?<br><br>Okay. Now I would like to talk about what we know about the experiences of young people with cancer who can't be cured, and who eventually die of their cancer. We know that, in their last few weeks of life, most of them experience a lot of medical care, like chemotherapy, time in the hospital or emergency room, and time in the intensive care unit. What do you think about that? Does that sound like the kind of care you would want, the kind of care you wouldn't want, or would it depend? Why?<br><br>We also know that many young people die in the hospital, although some die at home. Can you imagine what you would want? Why?<br><br>Are there any ways you would want to be sure that your life has had meaning?

© 2023 Mastropolo R et al. *JAMA Network Open*.

	We have talked about different priorities that you have, and also different priorities that other young people have. As you think about the many different things that are important to you, what do you think is most important for doctors and nurses to understand about you? If you could tell your doctor or nurse anything about what matters to you most, what would you tell him/her?<br><br>Is there anything else you would like for me to know about the things we have talked about? Are there other things that you would like to express about what might be important to people your age if they get to a point when treatment is no longer working?<br><br>Thank you for talking with me about these things. I know these are hard topics but I have learned so much from your perspective.

© 2023 Mastropolo R et al. *JAMA Network Open*.
