# Supplementary material for: Patient, Caregiver, and Clinician Perspectives on Core Components of Therapeutic Alliance for Adolescents and Young Adults With Advanced Cancer: A Qualitative Study
Source: JAMA Netw Open. 2023 Aug 9;6(8):e2328153. doi: 10.1001/jamanetworkopen.2023.28153 (PMC10413170; doi:10.1001/jamanetworkopen.2023.28153)
Supplement: Supplement 2. — Data Sharing Statement [file jamanetwopen-e2328153-s002.pdf]

Data Sharing Statement

Mastropolo. Patient, Caregiver, and Clinician Perspectives on Core Components of Therapeutic Alliance for Adolescents and Young Adults With Advanced Cancer. *JAMA Netw Open*. Published August 09, 2023. doi:10.1001/jamanetworkopen.2023.28153

Data
Data available: Yes
Data types: Deidentified participant data, Data dictionary
How to access data: Please contact the corresponding author for details of data sharing. Data will be shared in compliance with Dana Farber Cancer Institute and NIH policies.
When available: With publication

Supporting Documents
Document types: None

Additional Information
Who can access the data: Researchers whose proposed use of the data has been approved.
Types of analyses: Relevant analyses appropriate to the available data
Mechanisms of data availability: After approval of a proposal and with a signed data access agreement
Any additional restrictions: No additional restrictions
